# Lapatinib induces autophagic cell death and inhibits growth of human hepatocellular carcinoma

**DOI:** 10.18632/oncotarget.2045

**Published:** 2014-05-31

**Authors:** Yu-Jen Chen, Chih-Wen Chi, Wen-Chi Su, Huey-Lan Huang

**Affiliations:** ^1^ Department of Medical Research Mackay Memorial Hospital, Taipei, Taiwan; ^2^ Department of Radiation Oncology, Mackay Memorial Hospital, Taipei, Taiwan; ^3^ Institute of Traditional Medicine, School of Medicine, National Yang-Ming University, Taipei, Taiwan; ^4^ Institute of Pharmacology, Taipei Medical University, Taipei, Taiwan; ^5^ Research Center for Emerging Viruses, China Medical University Hospital, Taichung, Taiwan; ^6^ China Medical University, Taichung, Taiwan; ^7^ Department of Bioscience Technology, College of Health Science, Chang Jung Christian University, Tainan, Taiwan

**Keywords:** lapatinib, autophagy, hepatoma, cell death

## Abstract

Lapatinib, an orally adminstered small-molecule tyrosine kinase inhibitor targeting epidermal growth factor receptors (EGFR) and Her2/Neu, has been widely accepted in the treatment of breast cancer. In this study, we found that lapatinib induced cytotoxicity in human hepatoma Huh7, HepG2 and HA22T cells. For the mode of cell death, we found lapatinib induced a higher percent of dead cells and a lower percent of hypodiploid cells, suggesting non-apoptotic cell death in lapatinib-treated hepatoma cells. Moreover, lapatinib-induced autophagy in hepatoma cells was confirmed by the detection of autophagic LC3-II conversion, the up-regulation of autophagy-related proteins, and the down-regulation of p62 by immunoblotting. Autophagic cell death was demonstrated by images of punctuated LC3 patterns, a higher percent of acridine orange positive cells, as well as a partial rescue of cell death by autophagy inhibitor 3-methyladenine or chloroquine. We also found massive vacuoles in lapatinib-treated hepatoma cells by electronic microscopy. In addition, the shRNA of knocked-down autophagy-related proteins rescued the hepatoma cells from lapatinib-induced growth inhibition. We also demonstrated a reduction of tumorigenesis by lapatinib *in vivo*. In conclusion, lapatinib induced autophagic cell death and the growth of human hepatoma cells. Our study provides potential cancer therapies by using lapatinib as a treatment for hepatoma.

## INTRODUCTION

Hepatocellular carcinoma (HCC) has been categorized as one of the five most common cancers in the world in recent years. The incidence of HCC has dramatically increased in Western countries and has become more prevalent in Asian countries in the last decades due to chronic liver disease, chronic hepatitis or cirrhosis induced by hepatotropic viruses, aflatoxin and alcohol consumption [1]. Even with aggressive therapy, the 5-year survival rate of patients with advanced HCC remains less than 10%[2]. This poor prognosis is due to high recurrent and metastatic rates even after the use of current treatment modalities such as surgery, trans-hepatic artery chemoembolzation, radiofrequency ablation, radiotherapy, and multitarget tyrosine kinase inhibitors[2-4]. Clearly, the development of novel therapeutics against HCC is an urgent task.

Lapatinib is a reversible tyrosine kinase inhibitor (TKI) developed for targeted therapy by GlaxoSmithKline [5-6]. Its known targets are tyrosine kinase in the cytoplastic domain of epidermal growth factor receptor (EGFR or ErbB1) and epidermal growth factor receptor-2 (ErbB2 or Her2). Due to the high incidence of EGFR/Her2 signaling amplification associated with a poor prognosis in some types of human cancers such as breast cancer, lapatinib in combination with capecitabine has been approved by the Food and Drug Administration as a therapy for patients with Her2-overexpressing or metastatic breast cancer that failed to respond to anthracycline, taxane, or the previous targeting agent, anti-ErbB2 monoclonal antibody transtuzumab (Herceptin) [5-6]. The advantages of lapatinib treatment include the specificity of the EGFR family members, better applicability through oral administration, and tolerability of adverse effects with high compliance. Lapatinib is well accepted in ongoing preclinical or clinical applications for treatment of various solid tumors, including those of the breast, lung, HCC, head and neck, vulva, colon, prostate, ovarian and gastric cancers [5, 7-9]. Compared to trastuzumab, lapatinib treatment also shows higher activity in Her2-positive breast cancer patients with PTEN mutations [5, 10]. Although the phase II trials for lapatinib and HCC show a marginal benefit to a subgroup of patients without predictive markers yet characterized [7-8], the role of lapatinib in the treatment of HCC remains to be elucidated.

Many anticancer drugs work by induction of programmed cell death in tumor cells. Apoptosis is the first well-characterized programmed cell death. Its unique point is cells with condensed and fragmented chromatin. Besides apoptosis, many studies have recently focused on anticancer-drug-induced non-apoptotic cell death, such as autophagic cell death and necroptosis [11-12]. In cells, autophagy was originally found to be a strategy to accelerate material and energy recycling by the digestion of aged organelles with autophagy-specific lysosomes, also called autophagosomes. Some cancer cells use this kind of autophagy to increase proliferation under stress or in some nutrient-insufficient microenvironments [11, 13]. However, in recent years, studies found autophagosome-associated autophagic cell death as the second type of well-studied programmed cell death. Compared to apoptosis, the different features of autophagic cell death include massive autophagic vacuolization (double-membraned vacuoles) inside the cytoplasm and the absence of chromosome condensation and nuclear fragmentation. Except for autophagic vacuoles, the specific features of autophagic cell death also include beclin-1 (ATG6), ATG5, ATG12, or ATG7 involvement and LC-I to LC-II conversion. More papers discuss breaking the apoptosis rules we knew a decade ago. For example, apoptotic cascade molecules, such as FADD and caspase-8, are also involved in autophagy, as mentioned above, since recruitment of TNFR molecule caspase-8 and FADD with autophagy-related proteins ATG5, ATG12, and ATG16L were observed in the development (generation) of early autophagosomes in certain autophagy signal pathways [14-15].

Potential targets for anticancer therapy in HCC include the EGFR and Her2 overexpressed in HCC and directly implicated in hepatocarcinogenesis. Previous investigations indicate that EGFR is actively expressed in human HCC cells and EGF is required for the growth of HCC cells [4, 16-17]. In addition, Her2 is expressed in a significant number of HCCs, and may be an independent prognostic factor [18]. In this study, we chose human HCC cells as a model system for dissecting the mechanisms of lapatinib-induced HCC cell death to extend the application of lapatinib in HCC, such as that for the administration of single therapeutic as reported in phase II clinical trials [7-8], as well as combinatory treatment with other drugs or radiotherapy. We found the mechanism of lapatinib-induced cytotoxicity of HCC is autophagic cell death.

## RESULTS

### Cytotoxicity of lapatinib in HCC cells

**Figure 1:**
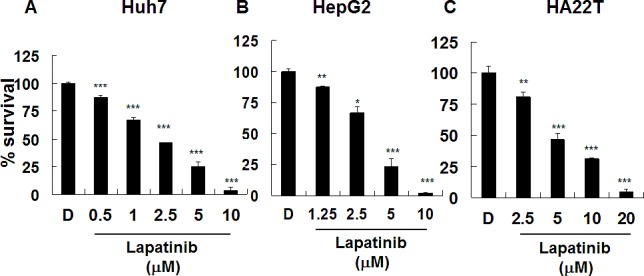
Inhibition of hepatoma cell proliferation by lapatinib Huh7 (A), HepG2 (B), or HA22T (C) HCC cells were left untreated or treated with 0.1% DMSO (vehicle, D) or with DMSO containing various concentrations of lapatinib (1.25, 2.5, 5, or 10 μM) for 3 days. Relative amounts of viable cells were detected by MTS assay. O.D. values from DMSO-treated control cells were designated 100%.

First, we tested lapatinib-induced cytotoxicity in several kinds of HCC cell lines, including well differentiated Huh7, HepG2, and poorly differentiated HA22T-VGH (HA22T) cells. Relatively viable cells were tested by a MTS [3-(4,5-dimethylthiazol-2-yl)-5-(3-carboxymethoxyphenyl)-2-(4-sulfophenyl)-2H-tetrazolium, inner salt] tetrazolium compound assay. As shown in Fig. 1, after 3 days of lapatinib exposure, the viability of these cells was dramatically reduced in a dose dependent manner when compared with the relative viability of cells treated with the same volume of vehicle dimethyl sulfoxide (DMSO). According to this assay, the EC_50_ for growth inhibition in lapatinib-treated Huh7, HepG2, and HA22T was about 2.11, 3.42 and 4.85 μM, respectively. In addition, we also detected the targets of lapatinib, EGFR and ErbB2, in these HCC cells (Fig. S1).

### Induction of non-apoptotic cell death by lapatinib in HCC cell lines

**Figure 2:**
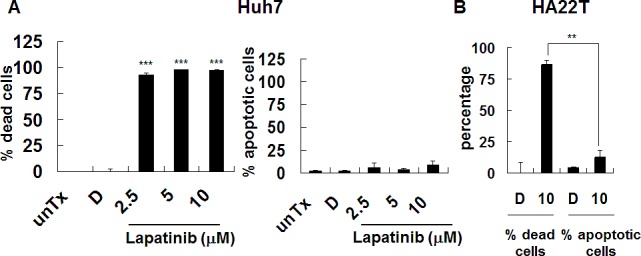
Induction of non-apoptotic cell death by lapatinib in HCC cells Cells were left untreated (unTx) treated, with 0.1% DMSO (vehicle, D) or 2.5–10 μM lapatinib for 3 days. After collection, cells were split into 2 tubes and resuspended in either PI containing PBS buffer (A or left panel of C, for detection of percent of total dead cells) or PI-containing hypotonic buffer (B or right panel of C, for detection of percent of hypodiploid or apoptotic cells) by flow cytometry.

As shown in Fig. 2, lapatinib-induced more than 90% or 80% of dead (PI-positive) Huh7 or HA22T cells (left panel), respectively; however, around or less than 10% sub G1 (hypoploid) cells (or DNA laddering, right panel) were detected [19-21]. We found the similar pattern for lapatinib-treated HepG2 cells (data not shown). We also used the Annexin V assay to detect externalized phosphotidyl serine to confirm the apoptosis assay (data not shown). To further elucidate lapatinib-induced cell death, we found the involvement of mitochondria in the lapatinib-induced death pathway due to the loss of mitochondrial integrity in HCC cells (Fig. S2). These data suggest mitochondria-involved non-apoptotic cell death induction (like autophagy or necrosis) by lapatinib in HCC cells, similar to the effect of lapatinib in K562 cells in previous investigations.

### Lapatinib-induced autophagic cell death in HCC cells

**Figure 3:**
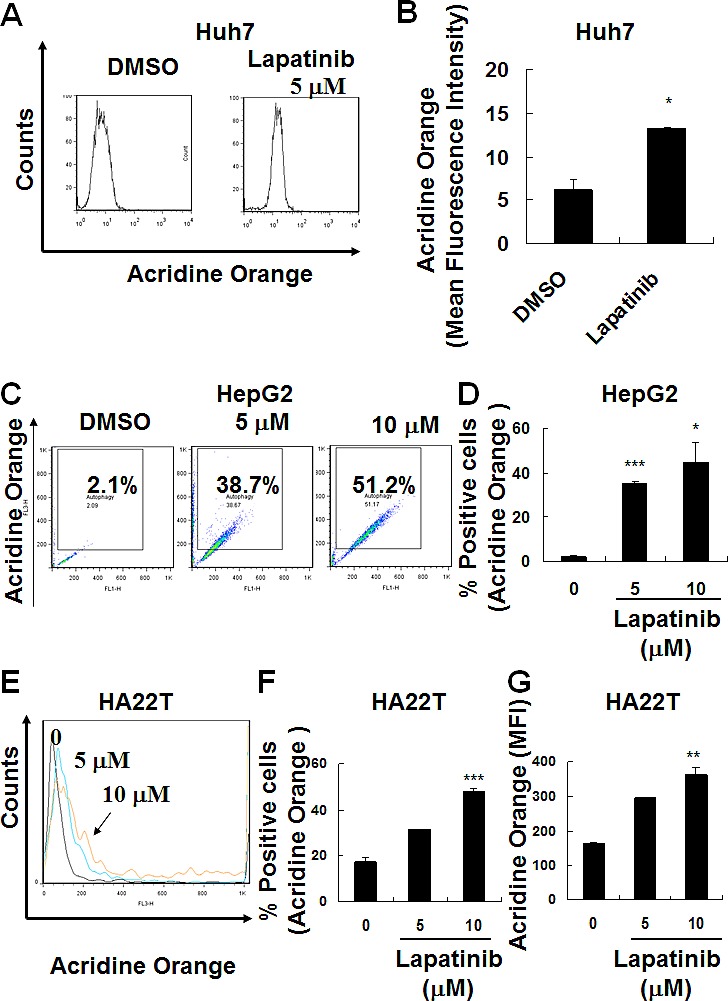
Induction of autophagy in lapatinib-treated HCC cells After DMSO, 5 or 10 μM lapatinib treatment for 3 days, Huh7 (A-B), HepG2 (C-D) and HA22T (E-G) HCC cells were stained with acridine orange, and the autophagic cells was analyzed by flow cytometry. Data are expressed as the mean fluorescence intensity (B or G) or percentage of acridine orange positive cells (C, D or F).

**Figure 4:**
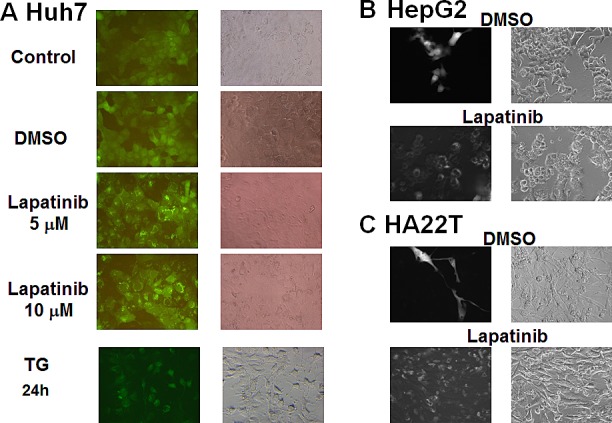
Induction of LC3 aggregations by lapatinib in HCC cells The stable expression of GFP-LC3 in HCC cells was prepared by the transduction of a GFP-LC3-containing lentivirus. After DMSO, 5 or 10 μM lapatinib or 150 nM thapsigargin (TG, positive control) treatment for 3 days; Huh7 (A), HepG2 (B), or HA22T (C) cells were observed by fluorescence microscopy.

Since we found lapatinib effective in killing HCC cells (near 100%, Fig. 2) with less than 10% attributed to apoptosis, we wondered if these cells could undergo autophagy or necrosis after lapatinib treatment. We tested whether lapatinib induced autophagic cell death in HCC cells by the detection of autophagic vacuoles-containing cells. As shown in Fig. 3, autolysosome formation (acridine orange positive cells) was detected after Huh7, HepG2 or HA22T cells were treated with lapatinib. In addition, punctuate LC3 aggregates (the evidence of LC3 conversion) were observed after 3 kinds of HCC cells were treated with lapatinib (Fig. 4)[22].

**Figure 5:**
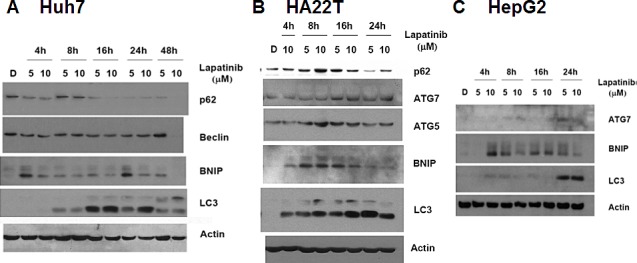
Induction of LC3 aggregations and autophagy-related genes by lapatinib in Huh7 (A), HA22T (B) and HepG2 (C) HCC cells. Cells were treated with DMSO or 5 or 10 μM lapatinib treatment for 4-48h as indicated. Cell lysates were then collected, subjected into 12% SDS-PAGE, and immunoblotted with antibodies against p62, Beclin, BNIP, ATG5, ATG7 LC3 and actin.

**Figure 6:**
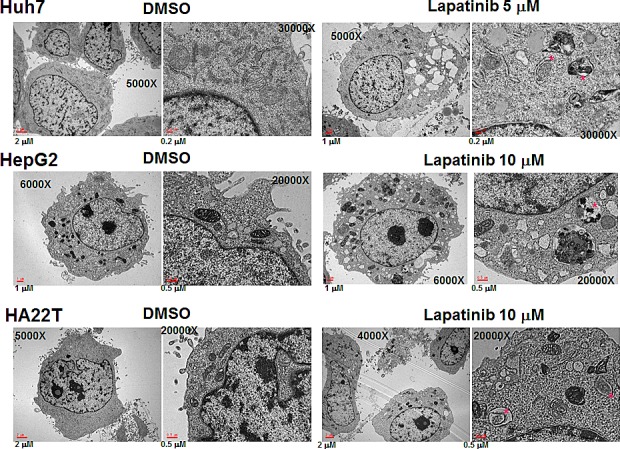
Microphotographs of lapatinib-treated HCC cells (A) After DMSO or 5 or 10 μM lapatinib treatment for 2 days, Huh7 (A), HepG2 (B) or HA22T (C) HCC cells were fixed, stained and observed by transmission electron microscopy. Mitochondria with vacuolization were marked with red dots. The magnification information is as indicated in each figure.

To further confirm lapatinib-caused autophagy in HCC cells, we checked the expression levels of some autophagy-related proteins in lapatinib-treated cells by immunoblotting [20]. As shown in Fig. 5, lapatinib induced LC3-II conversion, an increase of the autophagy-related proteins LC3 (LC3-II conversion), Beclin-1 (ATG6), ATG5, ATG7 and BNIP in HCC cells. Lapatinib also decreased the expression of p62 in Huh7 and HA22T cells. This is consistent with the degradation of p62 by autophagy[23]. We also checked the morphology of lapatinib-treated HCC cells by transmission electronic microscopy (TEM). As shown in Fig. 6, lapatinib induced a large amount of vacuoles in drug-treated HCC cells compared to the vehicle DMSO-treated cells. This phenomenon is specifically evident in both Huh7 and HepG2.

**Figure 7:**
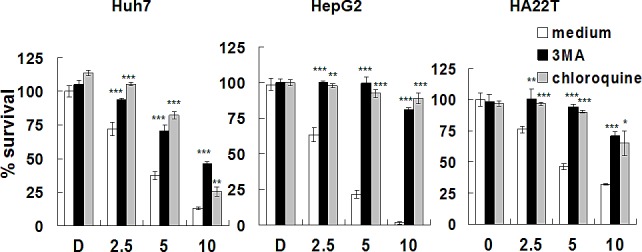
Rescue of Huh7, HepG2 or HA22T cells from lapatinib-induced cytotoxicity by the autophagy inhibitors Huh7, HepG2 or HA22T cells were left untreated or were treated with various concentrations of lapatinib in the presence or absence of 1.25-mM 3-MA or 5 μM chloroquine for 48h. Relative amounts of viable cells were detected using the MTS assay, and the relative percentage of growth inhibition was calculated as described in Fig. 1

To further confirm lapatinib-induced autophagic cell death, we tested the effects of two inhibitors, 3-methyladenine (3-MA) and chloroquine, which block the early stages of autophagic processes with a PtdIns3K inhibitor (3-MA) or a lysosomotropic compound for elevating pH in lysosomes/autophagosomes (chloroquine) [20]. Lapatinib-induced growth inhibition was effectively blocked when HCC cells were incubated with 3-MA or chloroquine (Fig. 7). This data suggests lapatinib induced autophagic cell death in HCC cells.

**Figure 8:**
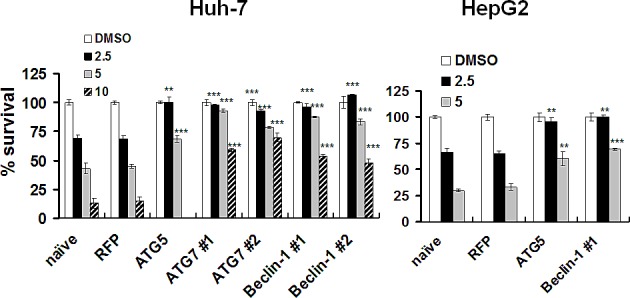
Protection of HCC cells from lapatinib-induced cytotoxicity by the knockdown of autophagy-related proteins. After transduction with shRNA expression lentivirus as indicated, Huh7 or HepG2 cells were selected and were treated with DMSO or 2.5 - 10 μM lapatinib for 48h, and then the relative percentages of growth inhibition were detected using the MTS assay and calculated as described in Fig. 1

To further test whether lapatinib induced autophagic cell death, we explored the role of autophagic proteins, such as ATG5, ATG7 and Beclin-1. In the vehicle- or lapatinib-treated cells, we knocked down expression of these autophagy-related proteins (Fig. S3) after transduction with a short hairpin RNA (shRNA) expression lentivirus system [21]. As shown in Fig. 8, the specific knockdown of ATG5, ATG7, and Beclin-1 mRNA, but not the non-targeting red fluorescent protein (RFP) mRNA, rescued the cells from lapatinib-mediated cell death. These data indicate, consistent with our previous figures, the involvement of these autophagic proteins with lapatinib-induced autophagic cell death in HCC cells.

### Lapatinib inhibits hepatoma tumor growth *in vivo* xenografts

**Figure 9:**
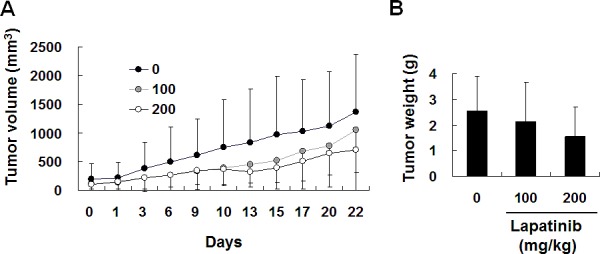
*In vivo* inhibition of HCC xenografts by lapatinib After inoculation of HepG2 cells (luciferase stably expressed) for 3 weeks, 12 mice were randomly divided into 3 groups and were orally administrated DMSO, 100 or 200 mg/kg of lapatinib for another 22 days (days 0-22). Tumor volumes were monitored every 2 days by detection of luciferase-activity-containing cells *in vivo* (A) or weighted after sacrificing on day 42 (B).

Since we have demonstrated the cytotoxicity effect of lapatinib in hepatoma cells, we wondered whether lapatinib treatment reduces HCC tumor growth *in vivo*. We used a xenograft system to inoculate human HepG2 cells into nude mice. As shown in Fig. 9, after being orally administered 100 or 200 mg/kg lapatinib for 3 weeks, lapatinib effectively reduced the tumor volumes in a dose dependent manner compared to the vehicle administration while lapatinib did not dramatically affect body weight and numbers of white blood cells in these mice (data not shown). This demonstrates a reduction of tumorigenesis by lapatinib *in vivo.*

## DISCUSSION

In this study, we clarified the mode of cell death and the mechanism of action of lapatinib against human HCC cells[24]. It could be regarded as translational information for the design of combinatory treatments with other regimens or radiotherapy for HCC.

The evidence of lapatinib-induced autophagic cell death includes a massive amount of dead cells with a limited amount of apoptotic cells (Fig. 2) [19-21]; the loss of mitochondrial integrity (Fig. S2)[25]; the detection of punctuate LC3 aggregates (Fig. 4) and acridine orange positive cells (Fig. 3)[22, 26]; the decreased expression of p62 and the elevated expression of autophagy-related proteins such as LC3, ATG5, ATG7, Beclin-1, and BNIP (Fig. 5); and the observation of massive vacuoles by TEM (Fig. 6) [11, 20, 27]. We also confirmed autophagic cell death by effectively reducing growth inhibition with both autophagy inhibitor 3-MA and lysosomal inhibitor chloroquine (Fig. 7) and by the expression of shRNA against autophagy-related proteins ATG5, ATG7 and Beclin-1 (Fig. 8)[11, 20-21, 27]. Most of all, we found a significant reduction of tumor growth in a dose-dependent manner *in vivo* after oral administration of lapatinib in a human HCC xenograft system (Fig. 9). We also found elevated E-cadherin expression in lapatinib-treated Huh7 HCC cells (Fig. S4), implicating the potential of lapatinib against the metastasis of HCC. This point will be further studied in the future.

According to our previous study, tumor cells are relatively sensitive to lapatinib as compared to normal counterparts like human CD14^+^ monocytes or mouse bone-marrow cells [21]. For attached cells, we also found lapatinib easily kills E1A/Ras transformated[28-29], but not its normal diploid counterpart, wild-type mouse embryonic fibroblasts with the same genetic background (data not shown). This correlates with the safety advantage for the administration of lapatinib to patients.

Although there are some reports about apoptotic cell death in lapatinib-treated cancer cells, autophagic cell death has also been induced in lapatinib-treated leukemia, HCT116 colon and bladder cancer cells[21, 30-31] and breast cancer cells co-treated with lapatinib and obatoclaxs[10], similar to our present study. Autophagic cell death is also induced by many other stimuli, such as platonin-treated leukemia cells[26], TNFα-treated cells, and death receptor-mediated cell death in cells lacking FADD, caspase activation, or NF-κB activation[15, 32-33].

Although unlike the obvious overexpression of ErbB2 in about 30% of breast or lung cancer patients[5], there are still accumulated studies demonstrating mutations or aberrant expressions of EGFR superfamily members in HCC, such as ErbB2 overexpression or truncated ErbB2 in a certain population of HCC patients [16-18, 34]. According to our data, we detected similar expression levels of EGFR and ErbB2 in our HCC cells (Fig. S1) as compared to A549 lung adenocarcinoma cells, which belong to cells expressing low levels of these receptors [9]. Since those human tumor cell lines with EGFR/ErbB2-overexpressing are more sensitive to lapatinib cytotoxicity, according to our study, lapatinib blocks growth signals mediated by either important EGFR/ErbB2 signaling through moderate amounts of EGFR and ErbB2 receptors or unveiled targets associated with HCC proliferation. Moreover, although HepG2 cells have lower EGFR and Her2 expression (Fig. S1), their lapatinib sensitivity is similar to Huh7 and HA22T (Fig. 1). Further work includes exploring the correlations between lapatinib and the mutations or amplifications of EGFR/ErbB2 and their downstream signaling in HCC cell proliferation [34]. This may further clarify the roles of EGFR/ErbB2 signaling in the development of HCC.

HCC cells were reported autophagy-defective with impaired autophagy responses[35]. Therefore, induction of autophagy or autophagic cell death might have a role in enhancing treatment responses for HCC.

Our study demonstrates lapatinib-induced cytotoxicity of HCC through the mechanisms of autophagic cell death. To extend the clinical application of lapatinib against HCC, our results could be a reference and translational information for the design of combinatory treatments with other regimens or radiotherapy. From point view of drug re-positioning by using lapatinib, the strategy about combination of lapatinib and other treatments or radiotherapy against hepatoma will be tested in the future.

## METHODS

### Tissue culture and agent treatments

Human hepatoma Huh-7, HepG2 and HA22T cells were cultured in Dulbeco's modified Eagles' medium (Gibco) supplemented with 10% fetal calf serum (Hyclone), 100 IU/ml of penicillin (Gibco), 100 mg/ml of streptomycin (Gibco), and 1% nonessential amino acid (Gibco). One thousand-fold stock solution of lapatinib was prepared by dissolving a ground lapatinib tablet (GlaxoSmithKline) in DMSO (Sigma). For lapatinib and 3-MA (Sigma) or chloroquine co-treatment experiments, 20-mM of 3-MA or 500-fold chloroquine stock solution was prepared by dissolving both drugs in a culture medium.

### Growth inhibition analysis

After drug treatments, the viability of the cells was measured using the MTS assay (Promega), according to the manufacturer's instructions. The optical density (OD) value of DMSO-vehicle treated cells was calculated as 100% viability.

### Flow cytometry

For simultaneous detection of both dead cells and apoptotic cells by flow cytometry (FACSCalibur, Becton Dickenson), HCC cells with intact plasma membranes (live cells) or with DNA laddering (hypodiploid or sub G1 phase) were detected by cell-cycle detection assay as previously described[20-21]. After treatment, all floating and attaching HCC cells were collected, split into 2 tubes, and resuspended in 1 μg/ml propidium iodide (PI, Sigma) containing either phosphate buffered saline (PBS) or hypotonic buffer (0.1% sodium citrate, 0.1% Triton X-100, and 5 μg/ml PI). The former was used to detect the percentage of total dead cells (PI-positive cells) without intact plasma membranes, while the latter was used to detect apoptotic (hypodiploid) cells [20-21]. To detect the percent of acridine orange positive cells, cells were collected, stained with 10 μg/ml acridine orange, and then analyzed by flow cytometry. All data measured by flow cytometry were analyzed using FlowJo software (Tree Star).

### Western blot analysis

After lapatinib treatment, HCC cells were collected and resuspended in a lysis buffer (Sigma) with protease inhibitors (Roche Biochemicals). Cell lysates with 30 or 50 μg of protein were subjected to 12% sodium dodecylsulfate-polyacrylamide gel (SDS-PAGE) electrophoresis. For immunoblotting, the following antibodies were used: actin (Sigma), ATG5 (Cell Signaling), ATG7 (Santa Cruz Biochemicals), Beclin-1 (or ATG6, Cell Signaling), LC3 (or ATG8, Abgent), and nucleoporin p62 (Genetax).

### Transmission electronic microscopy (TEM)

After treatment, HCC cells were collected from culture dishes using a PBS washing buffer and a Trypsin-EDTA detaching buffer. According to the manufacturer's instructions (Electron Microscopy Science), cells were fixed by 2.5% glutaraldehyde-containing cacodylate buffer (pH 7.4), stained with 1% osmium tetroxide, and then embedded in Spurr's resin after dehydration. The morphology of cells was then observed by TEM.

### Gene knockdown by shRNA expression system

As described previously [21], a lentivirus-based shRNA expression system from the RNAi Consortium (TRC) was used to knockdown the expression of the following genes: ATG5, ATG7, Beclin-1 and RFP (as non-targeting shRNA control). These pLKO.1-shRNA constructions were from the National RNAi Core Facility, Academia Sinica, Taiwan[21]. We used 2 different shRNA constructs (#1 and #2, as indicated in Figs. 8 and S3) for ATG7 and Beclin-1 (TRCN0000007584, TRCN0000007587, TRCN0000033549 and TRCN0000033552, respectively)[21]. Lentiviral production, viral infection and puromycin selection were performed as per TRC's instructions. Finally, cells were treated with lapatinib, and viability was tested by the MTS assay as described above.

### *In vivo* human tumor xenograft system

Nude mice experiments were done in the animal colonies of Mackay Memorial Hospital after approval by the Institutional Animal Experimentation Committee of the hospital. Human HepG2 cells that stably expressed luciferase were first enriched after transfection and puromycin selection. As described previously[36], after inoculation of HepG2 luciferase stably expressed cells (10^6^ in 0.1 ml PBS) into the right hind limbs of Balc/c nude mice for 3 weeks, 12 mice with similar tumor volumes were selected and randomly divided into 3 groups. They were then orally administered a vehicle (0.5% hydroxypropyl-methylcellulose with 0.1% Tween-80), or 100 or 200 mg/kg lapatinib in vehicle every day for the next 3 weeks (days 0-22). Tumor volumes were monitored every 2 days *in vivo* by detection of the luciferase activity of cells with Xenogeny IVIS 200 Imaging System (PerkinElmer). The mice were left untreated for days 23-42. After sacrificing at day 42, tumor volumes inside the mice were double-checked with *weight and* caliper measurements.

### Statistical analysis

All data gathered from triplicate (or more) experiments were expressed as means ± S.D. P values (*P < 0.05, **P < 0.01, ***P < 0.001) and were analysed by Student's *t*-test between lapatinib-treated and DMSO control cells or as indicated in each figure. Values of EC_50_ were calculated from MTS assay data at day 3.

## SUPPLEMENTARY FIGURES


