# Supplementary material for: Lapatinib induces autophagic cell death and inhibits growth of human hepatocellular carcinoma
Source: Oncotarget. 2014 May 31;5(13):4845–54. doi: 10.18632/oncotarget.2045 (PMC4148104; doi:10.18632/oncotarget.2045)
Supplement: Supplementary file 1 [file oncotarget-05-4845-s001.pdf]

Lapatinib induces autophagic cell death and inhibits growth of human hepatocellular carcinoma

Supplementary Material

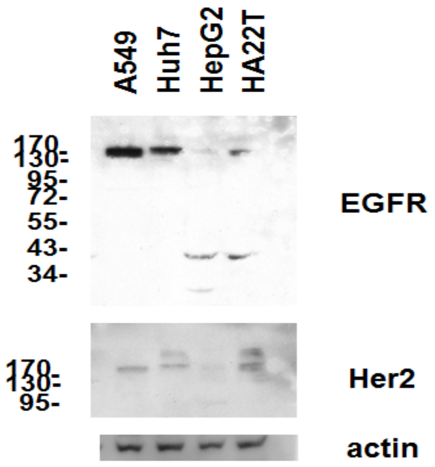

Figure S1: Expression of EGFR and ErbB2 in hepatoma cells. A549 lung adenocarcinoma and Huh7, HA22T and HepG2 HCC cell lysates were collected, subjected into 12% SDS-PAGE, and then immunoblotted with antibodies against EGFR, ErbB2 (Genetax) and actin.

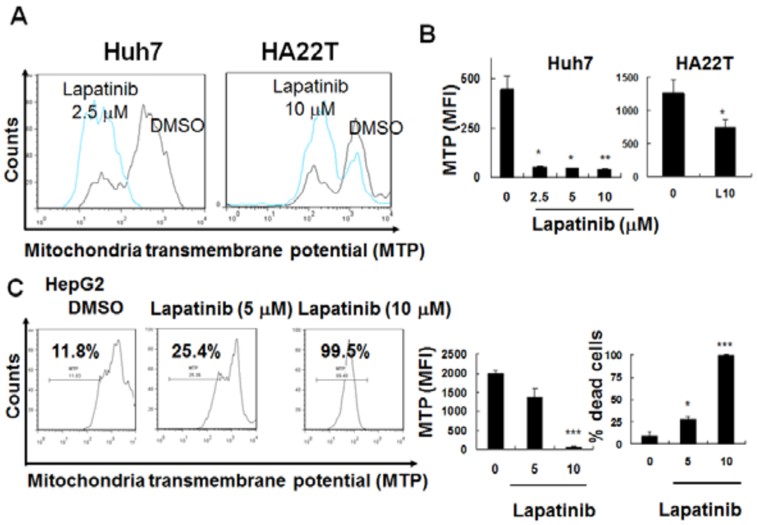

Figure S2: Lapatinib-induced loss of mitochondrial integrity in HCC cells. After DMSO, 2.5, 5, or 10 μM lapatinib treatment for 3 days, Huh7, HA22T (A) or HepG2 (C) HCC cells were stained with 3,3'-dihexyloxacarbocyanine iodide ($DiOC6_{(3)}$, Molecular Probes), a green fluorescent membrane dye. The mitochondrial transmembrane potential (MTP) of the cells was analyzed by flow cytometry, according to the manufacturer's instructions. Mean Fluorescence intensity(MFI) of MTP (B-C) or percentage of low MTP (dead) cells (C) is expressed as mean±SD.

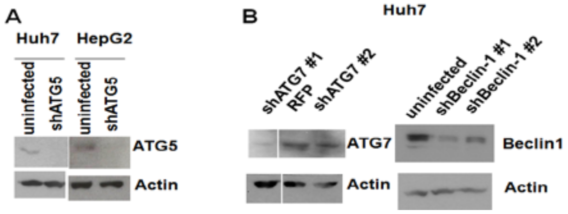

Figure S3: Detection of autophagy-related proteins in HCC cells after knockdown by a shRNA lentivirus expression system. After transduction with shRNA expression lentivirus as indicated, puromycin resistant Huh7 or HepG2 cells were selected. Cell lysates were then collected, subjected into 12% SDS-PAGE, and then immunoblotted with antibodies against ATG5 (A), ATG7, Beclin-1 (B) and actin.

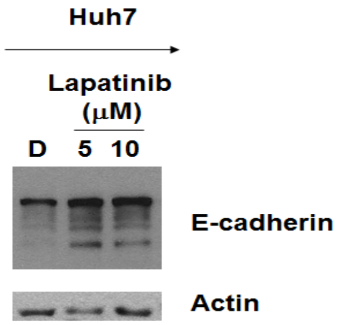

Figure S4: Inhibition of HCC metastasis by lapatinib. After DMSO, 5 or 10 μM lapatinib treatment for 3 days, Huh7 HCC cell lysates were collected and immunoblotted with antibodies against E-cadherin (Santa Cruz) and actin.
